# SARS-CoV-2 in Conjunctiva and Tears and Ocular Symptoms of Patients with COVID-19

**DOI:** 10.3390/vision5040051

**Published:** 2021-10-22

**Authors:** Teresa Rodríguez-Ares, David Lamas-Francis, Mercedes Treviño, Daniel Navarro, María Cea, María Jesús López-Valladares, Laura Martínez, Francisco Gude, Rosario Touriño

**Affiliations:** 1 Department of Ophthalmology, University Hospital of Santiago de Compostela, 15706 Santiago de Compostela, Spain; teresa.rodriguez@usc.es (T.R.-A.); maria.jesus.lopez.valladares@sergas.es (M.J.L.-V.); laura.martinez.perez2@sergas.es (L.M.); rosario.tourino.peralba@sergas.es (R.T.); 2 Faculty of Medicine, University of Santiago de Compostela, 15705 Santiago de Compostela, Spain; francisco.gude.sampedro@sergas.es; 3 Department of Microbiology, University Hospital of Santiago de Compostela, 15705 Santiago de Compostela, Spain; mercedes.trevino.castellano@sergas.es (M.T.); daniel.navarro.de.la.cruz@sergas.es (D.N.); maria.cea.pajaro@sergas.es (M.C.); 4 Clinical Epidemiology Service, University Hospital of Santiago de Compostela, 15706 Santiago de Compostela, Spain

**Keywords:** COVID-19, coronavirus, SARS-CoV-2, tears, conjunctiva, ocular surface

## Abstract

This study investigates the presence of SARS-CoV-2 in conjunctival secretions and tears and evaluates ocular symptoms in a group of patients with COVID-19. We included 56 hospitalized patients with COVID-19 in this cross-sectional cohort study. Conjunctival secretions and tears were collected using flocked swabs and Schirmer strips for SARS-CoV-2 reverse-transcriptase polymerase chain reaction (RT-PCR). Assessment of ocular surface manifestations included an OSDI (Ocular Surface Disease Index) questionnaire. Patients had been admitted to hospital for an average of 2.4 days (range 0–7) and had shown general symptoms for an average of 7.1 days (range 1–20) prior to ocular testing. Four (7.1%) of 56 conjunctival swabs and four (4%) of 112 Schirmer strips were positive for SARS-CoV-2. The mean E-gene cycle threshold values (Ct values) were 31.2 (SD 5.0) in conjunctival swabs and 32.9 (SD 2.7) in left eye Schirmer strips. Overall, 17 (30%) patients presented ocular symptoms. No association was found between positive ocular samples and ocular symptoms. This study shows that SARS-CoV-2 can be detected on the conjunctiva and tears of patients with COVID-19. Contact with the ocular surface may transmit the virus and preventive measures should be taken in this direction.

## 1. Introduction

SARS-CoV-2 coronavirus was first detected in December 2019 in the Chinese city of Wuhan and is known to cause Coronavirus Disease 2019 (COVID-19). Affected patients can develop symptoms, such as fever, cough, dyspnea, myalgia and gastrointestinal disorders. Severe cases present with bilateral pneumonia and hypoxemia, leading to acute respiratory distress syndrome (ARDS). Severe COVID-19 can affect multiple organs in the context of cytokine storms.

The main route of transmission of SARS-CoV-2 is through small respiratory droplets generated when an infected person coughs or sneezes, although it can also be transmitted through contaminated objects and facial contact [1]. This coronavirus has been detected in tears and conjunctival secretions, with higher positivity rates among patients with severe COVID-19 [2]. Recently, several studies have found ocular manifestations in patients with COVID-19. These findings suggest that ocular exposure could be a potential infection route and contribute to disseminating the infection through hand contact. The aim of the present study was to detect the presence of SARS-CoV-2 in the conjunctiva and tears of patients with COVID-19, as well as to describe their ocular symptoms.

## 2. Patients and Methods

We enrolled 56 hospitalized patients in this cross-sectional study carried out at the University Hospital of Santiago de Compostela, Spain. Patients were eligible if they had RT-PCR-confirmed SARS-CoV-2 infection (nasopharyngeal swab) and were within the first 20 days from the onset of symptoms. Patients admitted to the intensive care unit or who had a very poor general condition were excluded from this study. Written consent was obtained from all of the patients. The study was conducted in accordance with the guidelines of the Declaration of Helsinki and the principles of good clinical practice and was approved by the Institutional Review Board of the Galician Public Health Service (2020/228).

Clinical data were obtained from electronic medical records. Disease severity was classified as mild, moderate, or severe according to the guidelines published by the National Institutes of Health (NIH) [3]. Patients were asked about their ocular symptoms and the Ocular Surface Disease Index (OSDI) questionnaire was filled out.

Two methods were used to collect samples: (a) swabs of the tarsal conjunctiva and (b) Schirmer strips for tear collection. Conjunctival samples were taken with flocked swabs from the lower eyelid fornix of the right eye and were collected using liquid transport media for molecular and culture techniques (DeltaLab, Barcelona, Spain). The Schirmer test was performed without anesthesia by inserting paper filter strips (Standardized Schirmer tear test strips, Alcon, Fort Worth, TX, USA) in the inferior fornix, in both eyes. The moisturized length of the strip was measured after five minutes. Strips were put in sterile microcentrifuge tubes and sent to the laboratory for analysis. All samples were collected by the same Ophthalmologist and submitted to the same reference laboratory. Swab and Schirmer strip samples were stored at −80 °C until processing.

Prior to RNA extraction, samples were pretreated as follows. Conjunctival exudates were inactivated by incubation with external lysis buffer (Roche, Basel, Switzerland) for 15 min at room temperature. Each strip impregnated with tears was incubated with 1.5 mL of external lysis buffer (Roche) for 2 h with shaking, at room temperature. Viral RNA was extracted using the Magna Pure 24 automated System (Roche). Detection and identification of SARS-CoV-2 E (envelop), RdRP/S (RNA polymerase/Spike protein) and N (nucleocapsid) genes were performed with multiplex real-time PCR (Allplex 2019-nCoV assay, Seegene Inc., Seoul, Korea). All processes were carried out following the manufacturers’ instructions.

Descriptive data are presented as mean ± SD or percentages. A Chi-square test was used to determine whether there was an association between RT-PCR positivity from ocular samples and ocular symptoms.

## 3. Results

Fifty-six patients with RT-PCR-confirmed COVID-19 were included in the study. Thirty-two (57%) were male and 24 (43%) were female. The mean age was 69 years, ranging from 27 to 89 years. Disease severity was mild in five (8.9%) patients, moderate in 30 (53.6%) patients and severe in 21 (37.5%) patients.

Patients had been admitted to hospital for an average of 2.4 days (range 0–7) prior to ocular testing. The mean time from the onset of general symptoms until the collection of ocular samples was 7.1 days (range 1–20 days). The most common systemic comorbidity was hypertension, which was present in 48.2% of patients, followed by diabetes (28.6%), cancer (23.2%), heart disease (21.4%), obesity (17.9%) and chronic lung disease (16%). Nine (16.1%) patients did not have any relevant past medical history. Nineteen patients (33.9%) had previous ocular conditions, the most common being cataract surgery, in nine patients (16.1%), followed by glaucoma (5.4%), retinal vein occlusion (3.6%), strabismus (3.6%), uveitis (1.8%), refractive surgery (1.8%) and diabetic retinopathy (1.8%).

Four (7.1%) of the 56 conjunctival swab samples tested positive for SARS-CoV-2. Four (3.6%) of the 112 Schirmer strips were positive for SARS-CoV-2. In the four patients with positive conjunctival swab samples, Schirmer strip samples were negative in both eyes in one patient, positive only in the left eye in two patients and positive in both eyes in one patient. None of the patients had a negative conjunctival swab and a positive Schirmer strip sample. The characteristics of the patients with positive ocular samples are provided in Table 1. No association was found between the RT-PCR test from ocular samples and ocular symptoms. Twenty-nine percent of patients with a negative RT-PCR and 25% of patients with positive ocular RT-PCR had ocular symptoms (*p* = 0.679). Three of the patients had moderate illness and one presented a severe course of the disease.

Cycle threshold (Ct) values for each of the genes assayed (E, N, RdRP/S) are given in Table 2. The mean Ct values (E gene) were 31.2 (SD 5.0) in conjunctival swabs, 32.9 (SD 2.7) in left eye Schirmer strips and 24.2 (SD 8.2) in nasopharyngeal swabs. It must be noted that nasopharyngeal and ocular samples were not taken on the same day.

Tear production (Schirmer test) was higher in right eyes (mean 12.0 mm, SD 8.7 mm) than in left eyes (mean 7.5 mm, SD 5.3). Overall, 17 (30.4%) patients presented ocular symptoms (grittiness, 16.1%; ocular pain, 7.1%; photophobia, 1.8%; blurred vision, 3.6%; conjunctival hyperemia, 3.6%; itching, 3.6%; secretion, 7.1%). Nine patients with moderate disease and eight with severe disease had ocular manifestations. The average score of the OSDI questionnaire was 4.9 points (SD 8.6, range 0–30).

## 4. Discussion

In the present study carried out in hospitalized patients with COVID-19, we found a 7.1% prevalence of SARS-CoV-2 in conjunctival samples, which is comparable to the rates reported by other authors [2,4,5,6,7]. The SARS-CoV-2 virus had previously been detected in ocular tissues [8] and conjunctival symptoms have been observed in patients with COVID-19. The ocular surface is, therefore, thought to have a role as an entry point and reservoir for transmission of the virus.

In a meta-analysis of 2347 patients, Aggarwal et al. [9] reported an overall 3.5% SARS-CoV-2 positivity in conjunctival swabs. Other studies found variable rates, ranging from no positive samples [10] up to 24% positive [11]. A relatively low percentage of positive samples in the conjunctiva may be due to insufficient concentration for RT-PCR viral detection and/or a delay from the time of maximum viral load, which is usually in the first 5–6 days from the onset of symptoms. Seah et al. [10] collected most conjunctival samples between the second and third week after onset of symptoms and found no positive samples, whereas Arora et al. [11] delayed collection for a median of five days and found 24% positive samples. In our study, ocular sampling took place an average of 7.1 days from disease onset, and this may have influenced the number of positive cases we found. However, SARS-CoV-2 has been detected on the conjunctiva up to 27 days from disease onset, suggesting sustained viral replication [12].

Liang et al. [13] evaluated 37 patients with COVID-19 and found one positive conjunctival swab in a patient with severe COVID-19. Despite finding only one positive sample, these authors speculated that viral load in the conjunctiva could be linked to disease severity. Our results are in line with this hypothesis as our patients with positive ocular samples had moderate or severe disease.

In this study flocked swabs and Schirmer strips were employed for sampling. Most studies used flocked swabs for collecting conjunctival samples and RT-PCR for detecting viral genetic material. Some authors reported negative results when only Schirmer strips were used [10,14]. Arora et al. [11] found more positive samples when only swabs or a combination of swabs and Schirmer strips were used than when only Schirmer strips were used. In our series, we obtained four (7.1%) positive swab samples (of 56) and four (3.6%) positive Schirmer strips (of 112). These results suggest that swab sampling may be more efficient in detecting SARS-CoV-2. However we must remark that in our study swab samples were taken from the right eye prior to the strip samples, and this may have caused a reduction of the viral load. Fewer epithelial cells and secretions could be left, thereby reducing the possibility of detecting viral RNA with Schirmer strips. Other collection methods may be more sensitive than swabs and strips. Dutescu et al. [15] performed a lavage of the conjunctival sacs with saline and then collected the liquid with a laboratory capillary, obtaining 28% positive samples. We did not find any studies that used methods other than RT-PCR to demonstrate the presence of SARS-CoV-2 in ocular fluids, probably because no other methods were initially available or their efficacy in non-respiratory samples has not been fully studied.

Cycle threshold (Ct) values were high in ocular samples, indicating that the viral load was low on the ocular surface. The patient with positive conjunctival and tear samples in both eyes had a higher viral load in her eyes than the other patients, according to the Ct values. Interestingly, the only patient (#3) in whom the Ct values in ocular samples were lower than in the nasopharynx had more severe systemic disease.

Several reports have described ocular surface manifestations during SARS-CoV-2 infection in a variable percentage of patients [7,14,16,17]. The adaptive immune response generated by influenza or adenoviruses can lead to conjunctivitis or keratitis [18]. Coronaviruses do not seem to attract a strong response from the immune system and therefore, they are rarely associated with this type of reaction [19].

Another study analyzed 14 conjunctival samples from patients with COVID-19 who presented conjunctivitis, finding one positive sample with a high viral load [20]. Nevertheless, in line with our results, the positivity of conjunctival exudate or tear samples does not appear to be related to the presence of secretions or conjunctival inflammation. Although Xia et al. [6] only found positive samples among patients with ocular symptoms, other studies [7,21] reported the same rate of positivity in patients with or without conjunctivitis. Valente et al. [22] studied 27 children, most without ocular surface manifestations, of whom 11% had positive conjunctival samples. This confirms that SARS-CoV-2 RNA can be found on the unaffected ocular surface in patients with COVID-19, which can also be affected by other conditions, such as oxygen therapy or low humidity during hospitalization.

In our study, 69.6% of patients had a Schirmer test of 5 mm or less, and 30.4% of patients presented ocular symptoms. Our findings are similar to those described by Chen et al. [23], who reported 20.9% with dry eye, 12.7% with blurred vision and 11.8% with grittiness. Hong et al. [17] questioned patients after being discharged from hospital and found an increased OSDI score after hospitalization. However, Bozkurt et al. [24] did not find significant differences between a group of patients with COVID-19 and the control group regarding OSDI scores, Schirmer test, or TBUT (tear break-up time).

A meta-analysis of 1,167 patients with COVID-19 indicates that conjunctivitis could be a sign of the severity of the systemic disease [25]. In our study, 52.9% of patients with ocular surface manifestations had moderate disease, while 47.1% had severe illness. Patients with mild disease did not complain of ocular symptoms.

The transmission of SARS-CoV-2 through ocular secretions and tears of patients with COVID-19 is theoretically possible, on the basis of sample positivity and conjunctival cell tropism. Valente et al. [22] repeated the conjunctival sampling, which turned negative before nasopharyngeal swabs, suggesting that the viral load in the conjunctiva is lower. Deng et al. [26] argue that the risk of ocular transmission is limited, due to low positivity for SARS-CoV-2 in ocular samples. Rokohl et al. [27] did not find viral material on the ocular surface of 1145 hospitalized patients without COVID-19 infection. However, viral replication may potentially occur on the ocular surface, as ACE-2 receptors, which are expressed in lung epithelial cells, have also been found on the conjunctival epithelium [28]. Considering that the ocular surface is directly exposed to droplets and contaminated objects, it must be regarded as a possible route of transmission, and preventive measures should be taken in this direction [29]. Commercial ocular antiseptics may have a role in preventing ocular symptoms during COVID-19 infection, as they have shown to inhibit in vitro viral replication [30]. Eye care providers, in particular, must be aware of ocular transmission and follow strict disinfection protocols.

The main limitations of the present study are the small sample size and that nasopharyngeal and ocular samples were taken in different stages of the disease, which may affect the homogeneity of the results. We were not able to perform slit lamp examinations.

In conclusion, our data support the presence of SARS-CoV-2 in tears and conjunctiva of patients with COVID-19. Ocular transmission is possible and preventive measures should be taken in this direction.

## Figures and Tables

**Table 1:** Main characteristics of our four patients with positive conjunctival swab samples.

#	Age	Sex	Systemic Severity	Ocular Symptoms	OSDI	Schirmer LE	Conjunctival PCR	Strip PCR (RE)	Strip PCR (LE)	Days
1	83	F	Moderate	Pain, grittiness	20	5 mm	+	Neg	Neg	12
2	90	F	Moderate	None	0	5 mm	+	Neg	+	4
3	87	F	Severe	None	0	5 mm	+	Neg	+	2
4	75	M	Moderate	None	0	15 mm	+	+	+	10

Age is in years. OSDI: Ocular Surface Disease Index. Strip PCR: RT-PCR result from our Schirmer strip samples (RE: right eye; LE: left eye); Days: delay between onset of symptoms and ocular sampling; #: patient number; M: male; F: female; +: positive test; Neg: negative test.

**Table 2:** Cycle threshold (Ct) values of the RT-PCR assay for each patient samples/genes studied.

	Conjunct. Swabs			Schirmer RE			Schirmer LE			Nasopharynx		
#	E	N	RdRP/S	E	N	RdRP/S	E	N	RdRP/S	E	N	RdRP/S
1	37.8	37.7	37.8	Neg	Neg	Neg	Neg	Neg	Neg	25.0	25.5	24.8
2	28.8	27.2	29.4	Neg	Neg	Neg	31.4	30.8	30.1	15.0	17.1	15.6
3	32.1	33.3	31.7	Neg	Neg	Neg	36.0	36.1	36.5	34.8	34.7	35.0
4	26.3	26.0	26.8	32.3	32.8	34.1	31.4	32.1	33.1	22.2	23.8	23.1

E: Envelope gene; N: Nucleocapsid gene; RdRP/S: RNA-dependent RNA polymerase/Spike protein gene. Neg: negative RT-PCR test. Nasopharyngeal and ocular samples were not taken on the same day. #: patient number.

## Data Availability

Data available on request.
